# Microenvironment Promotes Tumor Cell Reprogramming in Human Breast Cancer Cell Lines

**DOI:** 10.1371/journal.pone.0083770

**Published:** 2013-12-30

**Authors:** Fabrizio D’Anselmi, Maria Grazia Masiello, Alessandra Cucina, Sara Proietti, Simona Dinicola, Alessia Pasqualato, Giulia Ricci, Gabriella Dobrowolny, Angela Catizone, Alessandro Palombo, Mariano Bizzarri

**Affiliations:** 1 Department of Experimental Medicine, Sapienza University of Rome, Rome, Italy; 2 Department of Surgery “Pietro Valdoni”, Sapienza University of Rome, Rome, Italy; 3 Department of Clinical and Molecular Medicine, Sapienza University of Rome, Rome, Italy; 4 Department of Basic and Applied Medical Sciences, University “G. D’Annunzio”, Chieti-Pescara, Italy; 5 Department of Experimental Medicine, Second University of Naples, Naples, Italy; 6 Department of Anatomy, Histology, Forensic Medicine and Orthopedics-Section of Histology and Medical Embryology, Sapienza University of Rome, Rome, Italy; 7 Centre of Space Bio-Medicine, University of Rome Tor Vergata, Rome, Italy; 8 Italian Space Agency (ASI), Rome, Italy; Spanish National Cancer Centre (CNIO), Spain

## Abstract

The microenvironment drives mammary gland development and function, and may influence significantly both malignant behavior and cell growth of mammary cancer cells. By restoring context, and forcing cells to properly interpret native signals from the microenvironment, the cancer cell aberrant behavior can be quelled, and organization re-established. In order to restore functional and morphological differentiation, human mammary MCF-7 and MDA-MB-231 cancer cells were allowed to grow in a culture medium filled with a 10% of the albumen (EW, Egg White) from unfertilized chicken egg. That unique microenvironment behaves akin a 3D culture and induces MCF-7 cells to produce acini and branching duct-like structures, distinctive of mammary gland differentiation. EW-treated MDA-MB-231 cells developed buds of acini and duct-like structures. Both MCF-7 and MDA-MB-231 cells produced β-casein, a key milk component. Furthermore, E-cadherin expression was reactivated in MDA-MB-231 cells, as a consequence of the increased *cdh1* expression; meanwhile β-catenin – a key cytoskeleton component – was displaced behind the inner cell membrane. Such modification hinders the epithelial-mesenchymal transition in MDA-MB-231 cells. This differentiating pathway is supported by the contemporary down-regulation of canonical pluripotency markers (Klf4, Nanog). Given that egg-conditioned medium behaves as a 3D-medium, it is likely that cancer phenotype reversion could be ascribed to the changed interactions between cells and their microenvironment.

## Introduction

For the last 50 years, the majority view about the carcinogenesis has centered almost exclusively on the somatic mutation theory (SMT) [1]. This theory claimed that “the problem of tumors is a cell problem” and that cancer was due to “a certain permanent change in the chromatin complex” which, “without necessitating an external stimulus, forces the cell, as soon as it is mature, to divide again.” [2]. According to SMT, cancer onset and development are events due to the accumulation of mutations in a few key-genes; therefore, when cancer begins, once the threshold has been crossed, there would be no way back towards normality. However, such framework is increasingly questioned by the accrual of paradoxical results [3]–[4].

Over a decade ago, Sonnenschein and Soto proposed the tissue organization field theory (TOFT), claiming that carcinogenesis takes place at the tissue level of biological organization, as does normal morphogenesis, and that chronic abnormal interactions between the mesenchyme/stroma and the parenchyma of a given organ, would be responsible for the appearance of a tumor [2]. Therefore, for the TOFT cancer is not a disease involving single cells, but different cell systems and their microenvironments; thus, carcinogenesis is a reversible process, whereby normal tissues (or their components) in contact with neoplastic tissues may normalize the latter [5]. A mounting body of evidence has suggested that re-establishment of appropriate interactions between human cancer cells and the surrounding microenvironment (i.e., stromal cells and the extracellular matrix) can reverse the neoplastic phenotype: indeed, these interactions play a crucial role in both cancer initiation and development, affecting gene transcription, differentiating and apoptotic pathways [6]–[10]. Normal cells located in the wrong tissue degenerate into cancer cells, whereas neoplastic cells introduced into a blastocyst, co-cultured with normal cells, implanted into a normal microenvironment or subjected to embryonic signals, either undergo apoptosis or become normal, thereafter contributing to the development of organised “normal” bodily structure [11]–[21]. In addition, embryonic or oocyte extracts, as an ex-ovo microenvironmental systems that program cell fate during development, are able to reverse tumorigenicity, through epigenetic modulation and activation of key-differentiating genes [22], [23], given that the oocyte environment provides all the factors necessary for turning differentiated nuclei into another state of differentiation [24].

We have previously shown that microenvironment derived from the albumen of unfertilized chicken eggs (EW, Egg White) dramatically modifies breast cancer cell architecture, and promotes the transition from a cancerous metabolomic profile (Warburg-like), towards an oxidative phenotype [25]. Recently we showed similar structural and behavioural changes also in TCam-2 human seminoma cells, where EW was able to modulate seminoma cell phenotype and behaviour, by ensuring a proper set of morphogenetic signals [26].

Herein we observed how EW could enact in MCF-7 and MDA-MB-231 breast cancer cells a complex differentiating process, as documented by both morphological and molecular changes. Moreover, we evaluated in MDA-MB-231 cells whether the EW-induced differentiating process can be considered a reprogramming process. According to Yamanaka cell reprogramming is conceived the process leading normal or cancer differentiated cells or cancer stem cells to become iPSCs (induced pluripotent stem cells), by inserting in the cell a set of four transcription factors (Oct4, Sox2, Klf4 and c-Myc), which have been demonstrated to be critical for staminality and cell differentiation [27]. Since then, different groups have reported the reprogramming of a number of solid tumors and derived tumor cell lines using all or some canonical genes, originally used by Yamanaka [28].

Herein, we tested whether the processes induced by EW in MDA-MB-231 cells can be strictly related to modulation of the endogenous activity of pluripotency genes Oct4, Sox2, Klf4, c-Myc, and Nanog.

## Materials and Methods

### Cell Lines

MCF-7 is a human breast cancer cell line isolated in 1970 from the malignant adenocarcinoma breast tissue. MCF-7 cells exhibit features of differentiated mammary epithelium: they are positive for epithelial markers such as E-cadherin, β-catenin and cytokeratin 18 (CK18) and they are negative for mesenchymal markers such as vimentin and α smooth muscle actin (α-SMA).

The MDA-MB-231 breast cancer cell line was obtained in 1973 at M. D. Anderson Cancer Center. The MDA-MB-231 breast cancer cells appear phenotypically as spindle shaped cells. MDA-MB-231 cells underwent epithelial-mesenchymal transition (EMT) that renders them prone to the metastasis; they are positive for mesenchymal markers such as vimentin and α-SMA and they express very weakly epithelial markers such as E-cadherin and CK18.

### Cell Culture

MCF-7 and MDA-MB-231 human breast carcinoma cell lines (ECACC, Sigma-Aldrich, St Louis, MO, USA) were cultured in Dulbecco modified Eagle’s medium (DMEM, Catalog n°EC B7501L; Euroclone Ltd., Cramlington, UK), supplemented with 10% Fetal Calf Serum (FCS, Euroclone), not essential amino acids and antibiotics (Penicillin 100 IU/ml, Streptomycin 100 µg/ml, Gentamycin 200 µg/ml, all from Euroclone). MCF-7 cells used for experiments were from passages 30 to 40, and MDA-MB-231 cells were from passages 5 to 15. The different number of passages for the two cell lines used in our experiments simply represents the passages performed in our laboratory from the purchase of each cell line.

### EW

The EW (Egg White) is the rough albumen derived from unfertilized chicken eggs, easily soluble in culture media. The hen eggs have been broken under a sterile hood and their content has been deposited in Petri dishes. The EW was separated from the yolk using a 20 ml syringe and then subjected to sterility testing. In all experiments EW was dissolved in DMEM to reach a final concentration of 10% of the volume. EW has been patented in Italy (patent number 0001400147) for its use in 2D and 3D cultures. The composition of EW has been reported by several authors; however, a compelling and exhaustive composition survey of the hen’s egg albumen still await to be defined, as new components have been recently identified, and others will presumably continue to be ascertained through the use of powerful new technologies [29].

### Ovalbumin

Ovalbumin (OV), the albumin from chicken egg white, is the major protein in EW, constituting about 54% of the total egg white protein [30]. We used OV as additional experimental condition to test its anticancer and differentiating effects. DMEM was supplemented with 10% FCS and with 8 mg/ml ovalbumin (A 5503, Sigma-Aldrich, St Louis, MO, USA), to reach a concentration of OV equal to that contained in DMEM 10% FCS +10% EW.

### 2D Cell Culture and Cell Proliferation Assay

MCF-7 and MDA-MB-231 human breast carcinoma cells were grown in culture plates in DMEM +10% FCS as a control condition, and DMEM +10% FCS+10% EW as experimental arm. Cultures were re-fed every second day. The duration of experiments varied from short times until 11 days, depending on the kind of analysis performed. For cell counting, every day the cells were trypsinized and suspended in IsoFlow™ Sheath Fluid electrolyte solution (Beckman Coulter, Inc. Fullerton, CA, USA). Cell count was performed by a particle count and size analyzer (Beckman Coulter, Inc. Fullerton, CA, USA) and by a Thoma hemocytometer. Similar experiments were performed using DMEM 10% FCS+OV as an additional experimental condition. Three replicate wells were used for each data point, and the experiments were performed six times.

### Optical Microscopy

MCF-7 and MDA-MB-231 cells were grown on six well tissue culture plate (Becton Dickinson Labware, Franklin Lake, NJ, USA) and they were photographed with Nikon Coolpix 995 digital camera coupled with Nikon Eclipse TS100 optical microscope (Nikon Corporation, Japan). Photomicrographs at different magnifications, were saved as TIFF files.

### Confocal Microscopy

MCF-7 and MDA-MB-231 cells were fixed in 4% paraformaldehyde for 10 minutes at 4°C, and incubated over night at 4°C with CMF 1,5% goat serum plus the following specific antibodies: anti-β-casein (sc-30041) and anti-β-catenin (sc-7963), all from Santa Cruz biotechnology. Cells were washed three times with PBS (1%BSA 0.2% Triton X 100) and incubated with the anti-mouse IgG-FITC PN IM1619, and anti-rabbit IgG-TRITC PN IM0834 secondary antibodies (Beckman-Coulter Inc. Fullerton, CA, USA), and TOPRO-3 iodide (Invitrogen, Eugene, Oregon, USA) to stain the DNA. Finally, cells were washed, mounted in Vecta Shield H-1000 (Vector Laboratories, Inc. Burlingame CA, USA), and analysed using a Leica confocal microscope (Laser scanning TCS SP2, Leica Microsystems Heidelberg GmbH, Mannheim, Germany) equipped with Ar/Ar/Kr and He/Ne lasers. The images were scanned under 40X oil objective. To determine the calibre of newly formed tubular structures in EW-treated samples, optical spatial series were performed as previously described [31].

### Electron Microscopy

MCF-7 cells, cultured with or without EW, were fixed in 2.5% glutaraldehyde in 0.1 M cacodylate buffer (pH 7.4), post fixed in 1% OsO4 in Zetterquist buffer, de-hydrated in ethanol, and embedded in epoxy resin. Ultrathin sections were contrasted in aqueous uranyl-acetate and lead-hydroxide, studied and photographed by a Hitachi 7000 Transmission Electron Microscope (Hitachi, Tokyo, Japan) as previously described [32].

### Western Blot

Cellular extracts and western blot protocols were performed as described elsewhere [19]. Blotted proteins were probed with the following antibodies: anti-β-casein (sc-30041), anti-β-catenin (sc-7963), anti-E-cadherin (sc-7870), anti-oct-3/4 (sc-5279), anti-ZEB1 (sc-10570), anti-ZEB2 (sc-18392), and anti-c-myc (sc-40) all from Santa Cruz Biotechnology; anti-α-tubulin (T5168) from Sigma; the anti-Histone H3 trimethyl Lys 4 antibody (39159) from Active Motif; the anti-Histone H3 antibody (ab1791), anti-klf4 (ab75486), anti-sox2 (ab97959), anti-nanog (ab80892) and the anti- α-SMA (ab5694) were from Abcam; the anti-LSD1(#2139), anti-Snail (#3879) and anti-CK18 (#4548) antibodies were from Cell Signaling. Antigens were detected with enhanced chemoluminescence kit (Amersham Biosciences, Little Chalfont Buckingamshire, England).

### DNA Extraction, Methylation Analysis and Cdh1 Transcript Analysis

Genomic DNA was isolated from cell lines by standard phenol and chloroform extraction with ethanol precipitation. The *cdh1* gene encodes E-cadherin. Methylation patterns within CpG island in exon 1 of the *cdh1* gene (sequence, 2126 bp to 1144 bp relative to transcription start; GenBank Accession No. D49685) were determined after the chemical modification of genomic DNA with EpiTect® Bisulfite Kit (QIAGEN GmbH, Hilden, Germany). A nested-PCR approach was used. In the first round of PCR, 100 ng of bisulfite-treated DNA was amplified using sequencing primers. The sequencing primers were 5′-GTTTAGTTTTGGGGAGGGGTT-3′(sense) and 5′-ACTACTACTCCAAAAACCCATAACTAA-3′ (antisense), and the cycling conditions consisted of 35 cycles of 94°C for 1 min, 50°C for 1,5 min, and 72°C for 1,5 min. The Taq DNA polymerase used was from Sigma-Aldrich. The size of the product following this initial PCR reaction was 270 bp. For the second round of PCR, this product was diluted 1∶50 in water, and 2 µl of the dilution were used for Methylation Specific PCR (MSP). Nested primer sequences for E-cadherin were 5′-TGTAGTTACGTATTTATTTTTAGTGGCGTC-3′ (sense) and 5′-CGAATACGATCGAATCGAACCG-3′ (antisense) for the methylated reaction and 5′-TGGTTGTAGTTATGTATTTATTTTTAGTGGTGTT-3′ (sense) and 5′-ACACCAAATACAATCAAATCAAACCAAA-3′ (antisense) for the unmethylated reaction. PCR parameters were as above, except that the annealing temperatures for the methylated and unmethylated reactions were 64 and 62°C, respectively. The product sizes of the methylated and unmethylated reactions were 112 and 120 bp, respectively.

Total RNA was isolated using TRI Reagent® (Sigma-Aldrich), treated with DNase I Amplification Grade (Invitrogen), and reverse-transcribed using ImProm-II™ Reverse Transcription System (Promega). Real Time PCR was performed using the ABI PRISM 7500 SDS (Applied Biosystems, USA), and Taqman® MGB probe Assay ID: Hs01023894_m1* (CDH1 gene) for E-cadherin and the Applied Biosystems® Human GAPD (GAPDH) Endogenous Control. The relative level for CDH1 gene was calculated using the 2^−ΔΔCt^ method and reported as fold increase versus control condition [33].

### Densitometry and Statistical Analysis

Western-blot images were acquired and analyzed through Imaging Fluor S densitometer (Biorad-Hercules). Optical density (O.D.) of each condition was normalized versus the signal of internal control α-tubulin. Data were expressed as mean (SD). The experiments for western blot analysis were repeated at least three times. Data from cell count and western blot were analysed with the Student’s unpaired *t* test.

Data from Real Time PCR were expressed as mean (SEM), and were analysed with the Mann Whitney test. The experiments for Real Time PCR were repeated six times. Differences were considered significant at the level of p<0.05. Statistical analysis was performed by using GraphPad Instat software (GraphPad Software, Inc.; San Diego, CA, USA).

## Results

### Cell Proliferation

**Figure 1:**
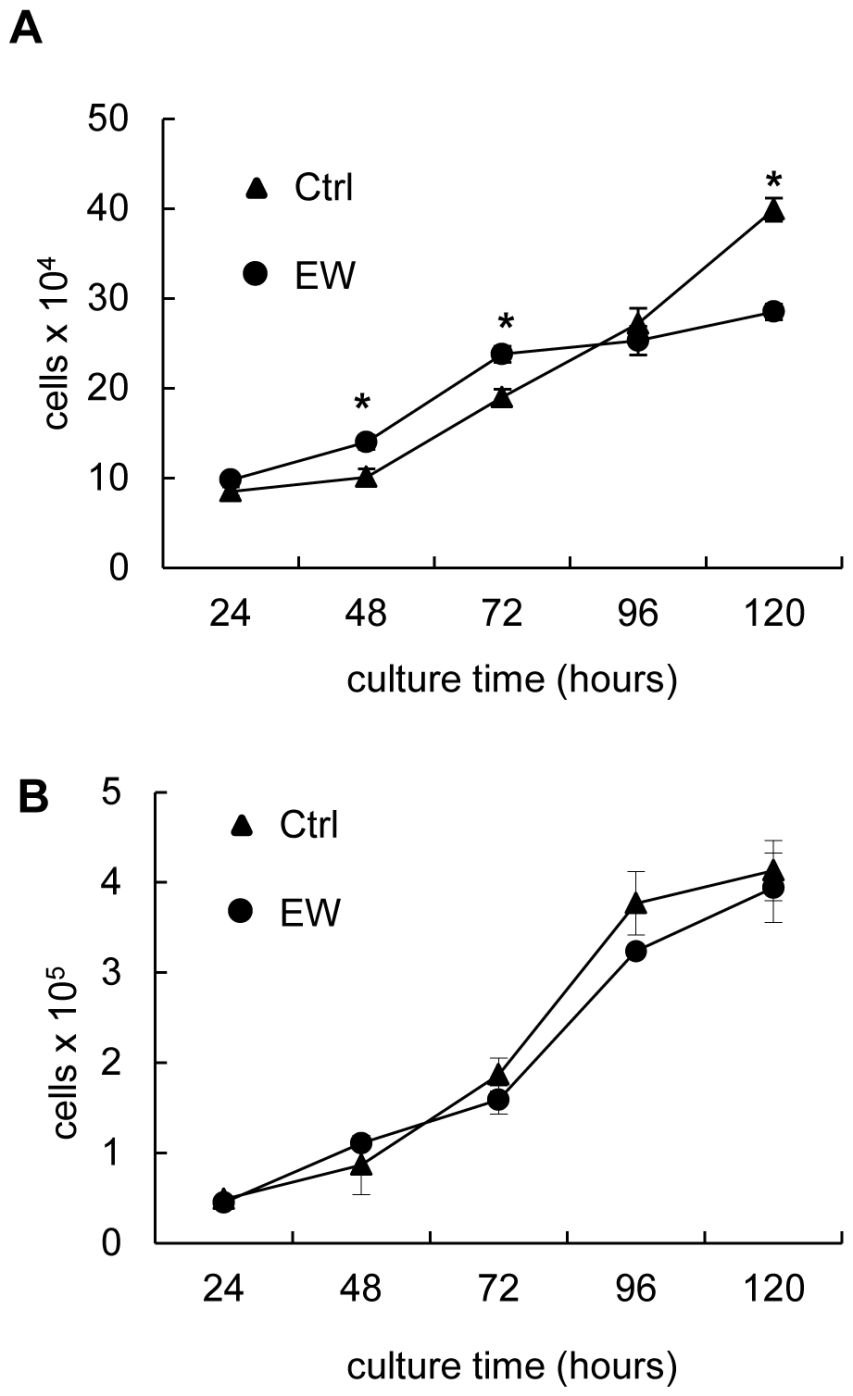
The growth of MCF-7 and MDA-MB-231 cells. Growth curves of MCF-7 (A) and MDA-MB-231 cells (B) in control condition (ctrl) and in EW treatment. The results are the mean (SD) of six independent experiments performed in triplicate; *p<0.001 vs ctrl by Student’s *t* test.

For both cell lines we did not find significant differences in the rate of apoptosis between the control and EW condition (not shown). EW-treated MCF-7 cells grew more rapidly with respect to control until the third day of culture. After 3 days (72 hours) of EW-treatment, MCF-7 cells were less numerous compared to the control (Fig. 1A); no significant differences in cell number have been noticed in MDA-MB-231 cells treated with EW medium (Fig. 1B).

### Mammary Gland Structures Development

**Figure 2:**
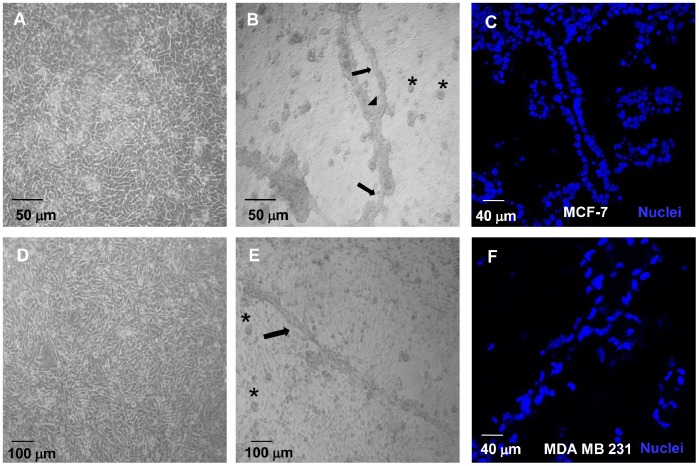
EW induces acini and duct-like structures development in 2D culture. Phase contrast microscopy. (A): confluent MCF-7 cells in control condition; (B) MCF-7 treated with EW (day 7); (D): confluent MDA-MB-231 cells in control condition; (E) MDA-MB-231 cells treated with EW (day 7). EW-treated cells formed acini (*) and duct-like structures (black arrows) that branched at specific points (black arrow head) and are resting on the monolayer of confluent cells. Confocal microscopy: (C), (F), representative optical sections at central level of acini and duct like structures formed after seven days of treatment with EW. The position of cell nuclei (blue) shows the cavitation of acini and duct-like structures formed in MCF-7 cells (C) and the cavitation of duct-like and acini buds (F) formed in MDA-MB-231 cells.

**Figure 3:**
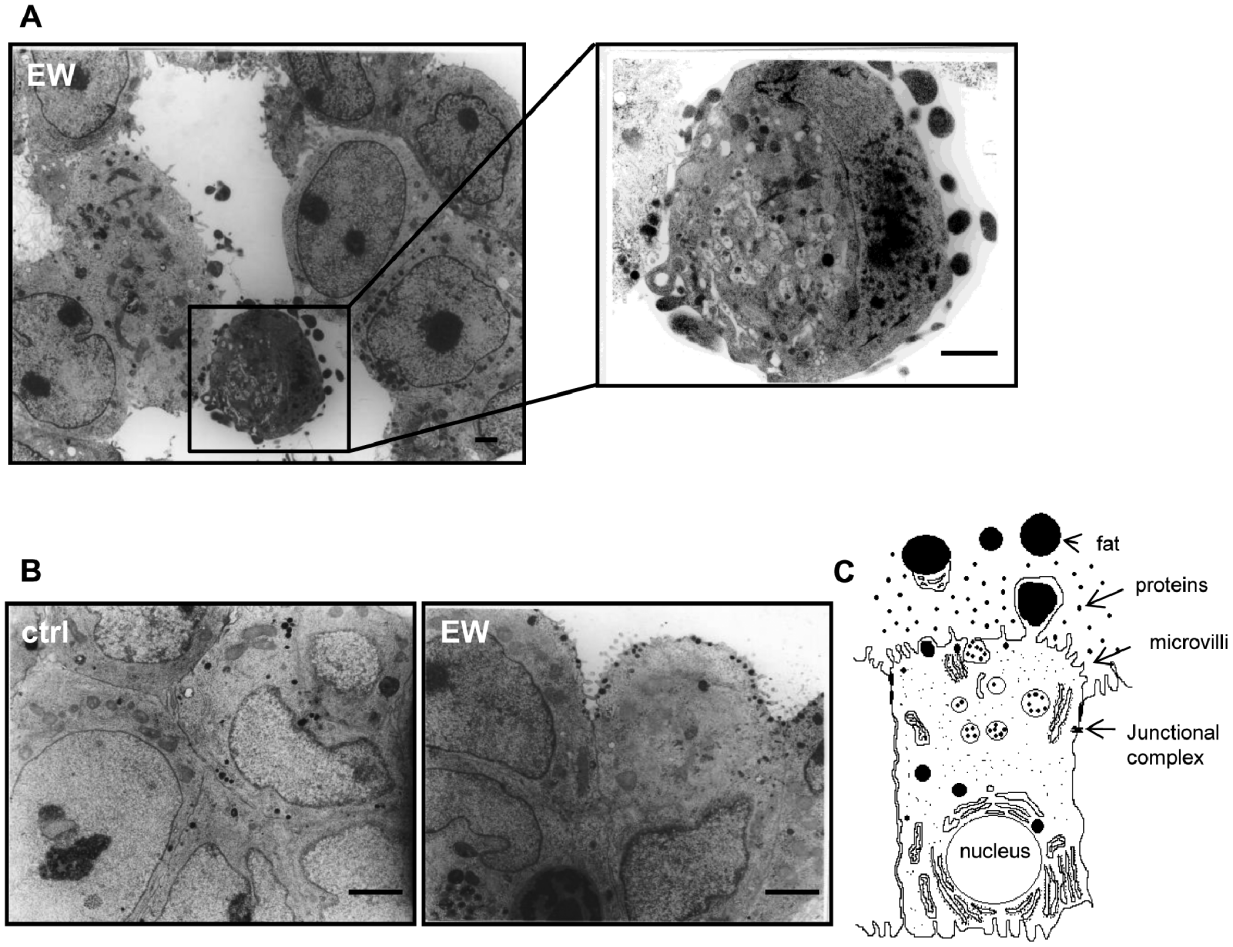
EW induces cell polarization in MCF-7 cells. TEM photomicrographs of MCF-7 cells at seven days of culture. (A) Ultrastructure of an acinus formed by EW-treated MCF-7 cells (day 7). The magnification of the insert shows a luminal cell undergoing cell death. Scale bars = 1 µm (B) The left image shows the morphology of the cell in control condition; the right image shows the cells treated with EW; Bars = 1 µm. The EW-treated cells were polarized and assumed the morphology of secreting cells with the nucleus in basal position and secretory vesicles in the apical portion of cell reach in microvilli, as depicted in (C).

In 2D culture, in EW-treated MCF-7 cells we observed the formation of hollow acini and duct-like bodies, akin to mammary gland structures (Fig. 2A–2C). Similarly, buds of acini and duct-like bodies were observed in MDA-MB-231 cells cultured with EW (Fig. 2D–2F). Tubular structures formed on EW-treated MCF-7 cells monolayers have mainly calibres ranging from 30 to 60 micron, whereas occasionally it is also possible to observe structures with calibres of about 200 micron. Cavitation processes, mainly driven by inner cell’s death, are required to ensure lumen formation. Cavitation of *de-novo* formed structures was evaluated by means of confocal microscopy, evidencing that three-dimensional structures, developed under EW culture, are hollow. A spatial series of optical sections relative to the formation of hollow duct-like structures in MCF-7 cells, detected with the staining of a membrane perimeter protein (such as β-catenin, in green), and of nuclei (blue), is reported in video S1 (Supporting Information). These data have been confirmed by transmission electron microscopy (TEM), showing cell death processes within the branched structures developed in EW-treated MCF-7 cells (Fig. 3A).

### Cell Polarization and β-casein Biosynthesis

**Figure 4:**
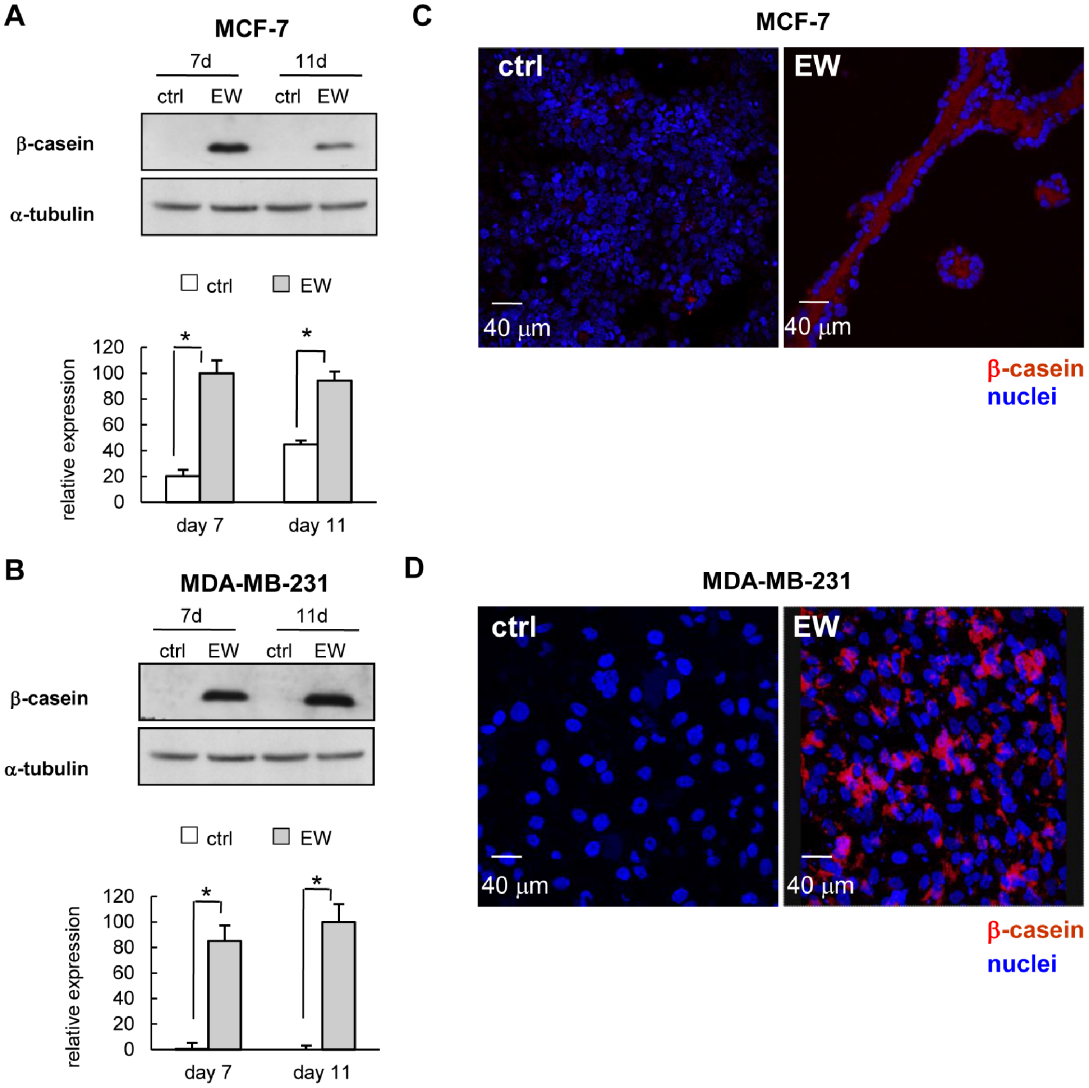
EW induces β-casein biosynthesis. (A) and (B): representative western blot showing the levels of the protein β-casein in MCF-7 and MDA-MB-231 cells respectively, after 7 and 11 days of treatment with EW. For each cell line is reported the densitometric quantification of β-casein, obtained normalizing the O.D. of protein bands versus the O.D. of α-tubulin bands, chosen as loading control. The results are the mean (SD) of three independent experiments; *p<0.001 vs ctrl by Student’s *t* test. Confocal microscopy: (C) and (D), representative optical sections of MCF-7 and MDA-MB-231 cells in control condition (ctrl) and after seven days of treatment with EW. (C) The immunostaining for β-casein (red) and cell nuclei (blue) shows the absence of β-casein in control (ctrl) cells and the presence of β-casein in the lumen of acini and duct-like structures in EW-treated MCF-7 cells. (D) Control (ctrl) cells are negative, whereas MDA-MB-231 cells treated with EW are positive for β-casein staining (red). Cell nuclei are stained in blue.

Epithelial breast cancer cells lose their polarization; on the contrary, EW-treated MCF-7 cells assume well polarized and typical secreting cell morphology (Fig. 3B and 3C). That finding is mirrored by the *de novo* biosynthesis of β-casein, the core component of milk. Indeed, whereas β-casein was not detected in control cells, high β-casein levels were observed in EW-treated cells after 7 and 11 days (Fig. 4A, and 4B). As documented by confocal microscopy, the secretion of β-casein is correctly oriented towards the lumen of acini and ducts (Fig. 4C, video S2 in Supporting Information). *De novo* β-casein biosynthesis has been found in MDA-MB-231 cells too, where confocal micro-pictures showed a strong signal for β-casein staining (Fig. 4D).

### E-cadherin Expression

**Figure 5:**
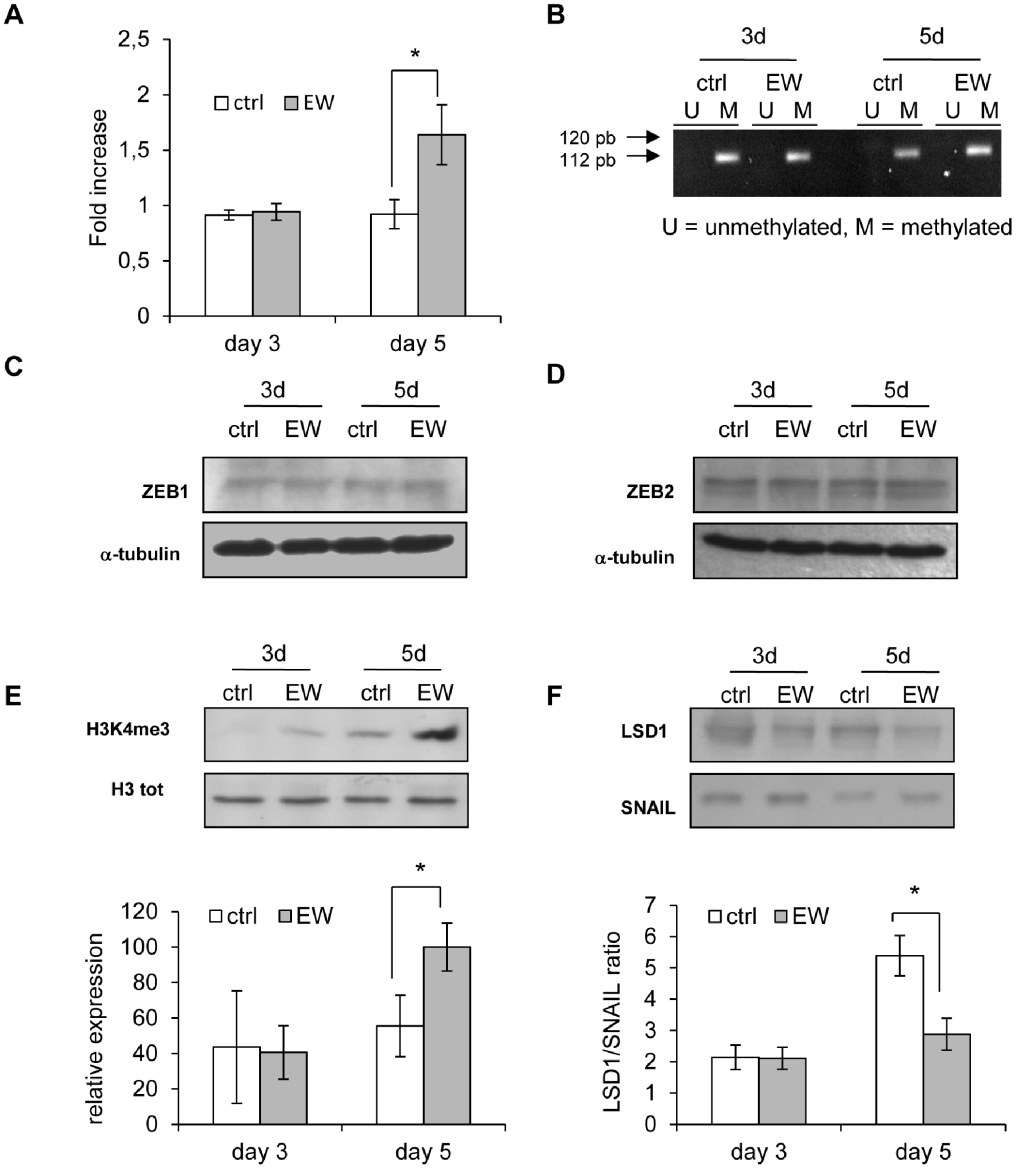
Cdh1 gene expression and its regulation. (A) Real-time PCR for Cdh1 gene in MDA-MB-231 cells treated for 5 days with EW. The results are the mean (SEM) of six independent experiments; *p<0.05 vs ctrl by Mann Whitney test. (B) Methylation of the Cdh1 gene promoter in MDA-MB-231 cells at 3 and 5 days of treatment with EW. Visible PCR product in lanes U indicates the presence of unmethylated sequences; visible PCR product in lanes M indicates the presence of methylated sequences. (C) and (D), representative western blot showing the levels of ZEB1 and ZEB2 proteins in MDA-MB-231 cells after 3 and 5 days of treatment with EW; proteins signals are shown together with α-tubulin bands, chosen as loading control. (E) Representative western blot showing the levels, after 3 and 5 days of treatment with EW, of the histone H3 trimethylated at lysine 4 (H3K4me3), and the levels of the total histone H3 (H3 tot) in MDA-MB-231 cells. Below is shown the densitometric quantification of H3K4me3, obtained normalizing the O.D. of protein bands versus the O.D. of Histone H3. (F) Representative western blot showing the levels, after 3 and 5 days of treatment with EW, of LSD1 and SNAIL proteins in MDA-MB-231 cells. Below is shown the LSD1/SNAIL ratio, obtained normalizing the O.D. of LSD1 bands versus the O.D. of SNAIL. The results are the mean (SD) of three independent experiments; *p<0.05 vs ctrl by Student’s *t* test.

*Cdh1* gene codifies for E-cadherin and it is very weakly expressed in MDA-MB-231 cells, being its promoter methylated [34]. In MDA-MB-231 cells exposed to EW, starting from the fifth day, the *cdh1* gene expression was significantly increased (Fig. 5A), without any detectable change on the methylation status of the *cdh1* promoter CpG island, recorded through the MSP assay (Fig. 5B). Yet, we cannot exclude the possibility that demethylation changes in *cdh1* promoter occurred at specific individual sites not captured by MSP assay.

Mechanisms other than CpG island methylation may be involved in the up-regulation of *cdh1* gene.

The involvement of the microRNA-200 family in *cdh1* regulation, through direct targeting of transcriptional repressors, such as ZEB1 and ZEB2, has been recently highlighted [35]. Hence, ZEB1 and ZEB2 levels were investigated, but without highlighting any significant differences in both control and EW-treated MDA-MB-231 cells (Fig. 5C and 5D).

**Figure 6:**
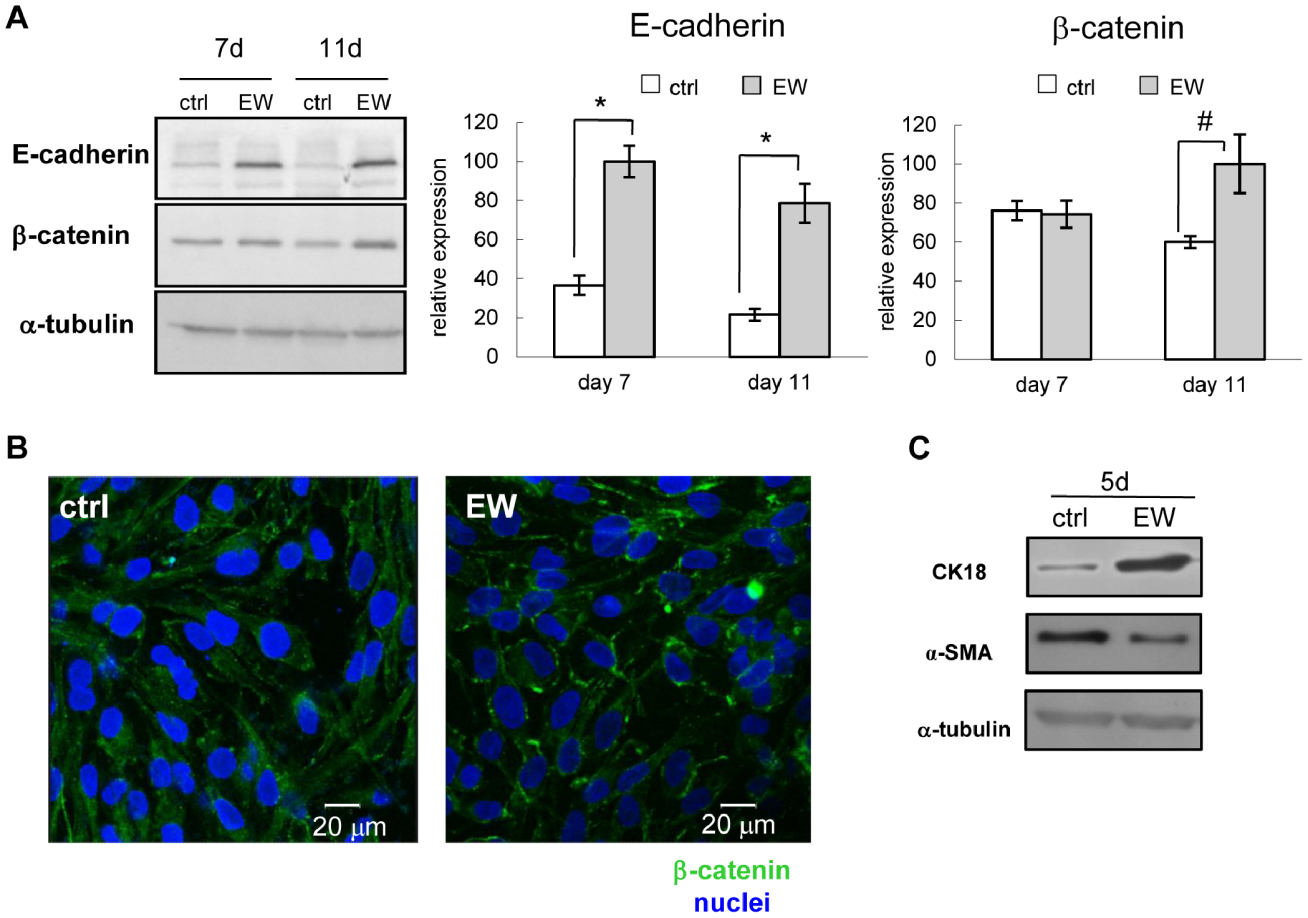
E-cadherin expression and β-catenin localization are influenced by EW. (A) Representative western blot showing the levels of the proteins E-cadherin and β-catenin in MDA-MB-231 cells, after 7 and 11 days of treatment with EW. Alpha tubulin was used as a loading control. At the right of the panel are reported the densitometric quantification of E-cadherin and β-catenin respectively, obtained normalizing the O.D. of protein bands versus the O.D. of α-tubulin bands, chosen as loading control. The results are the mean (SD) of three independent experiments; *p<0.001 and ^#^p<0.05 vs ctrl by Student’s *t* test. (B) Confocal microscopy of one representative optical section of MDA-MB-231 cells at the seventh experimental day: the immunostaining for β-catenin (green) shows a dispersed and punctuated staining in control condition (ctrl) with respect to the stronger staining that localized near the membrane perimeter for EW-treated cells. (C) Representative western blot showing the levels of the proteins CK18 and α-SMA in MDA-MB-231 cells, after 5 days of treatment with EW. Alpha tubulin was used as a loading control.

Furthermore, histone H3 trimethylation at lysine 4 may be associated with the transcriptional activation of the *cdh1* gene. Indeed, the extent of histone H3 trimethylation at Lys 4 in EW-treated MDA-MB-231 cells was 1.8 folds greater than in MDA-MB-231 control cells at fifth day (Fig. 5E). The Lysine-specific demethylase 1 (Lsd1) and SNAIL proteins are two crucial factors of a multiprotein complex that regulates histone H3 methylation status. When SNAIL is present the ratio Lsd1/SNAIL may be thought as a marker of the availability of this histone demethylating complex. This ratio significantly decreased with EW after the fifth day of treatment (Fig. 5F). Overall, these findings may explain the observed increase in E-cadherin protein biosynthesis, starting from the fifth day (Fig. 6A).

### β-catenin Localization

MCF7 cells did not undergo EMT, hence, as expected, no differences in the β-catenin membrane localization have been recorded in both EW-cultured MCF7 and controls (not shown).

In EW-cultured MDA-MB-231 cells the biosynthesis of β-catenin was slightly increased (Fig. 6A), and, even more importantly, β-catenin localization was shifted from nucleus and cytoplasm (where it acts as transcriptional regulator in cell proliferation), to the inner membrane, where β-catenin contributes to cytoskeletal architecture in epithelial differentiated cells (Fig. 6B) [36].

### CK18 and α-SMA Proteins

MDA-MB-231 cells express very weakly epithelial markers such as CK18 and they are positive for mesenchymal markers such as α-SMA. We found the increase of CK18 and conversely the decrease of α-SMA in EW-treated MDA-MB-231 cells with respect to control cells after 5 days of treatment (Fig. 6C).

### Pluripotency Markers

**Figure 7:**
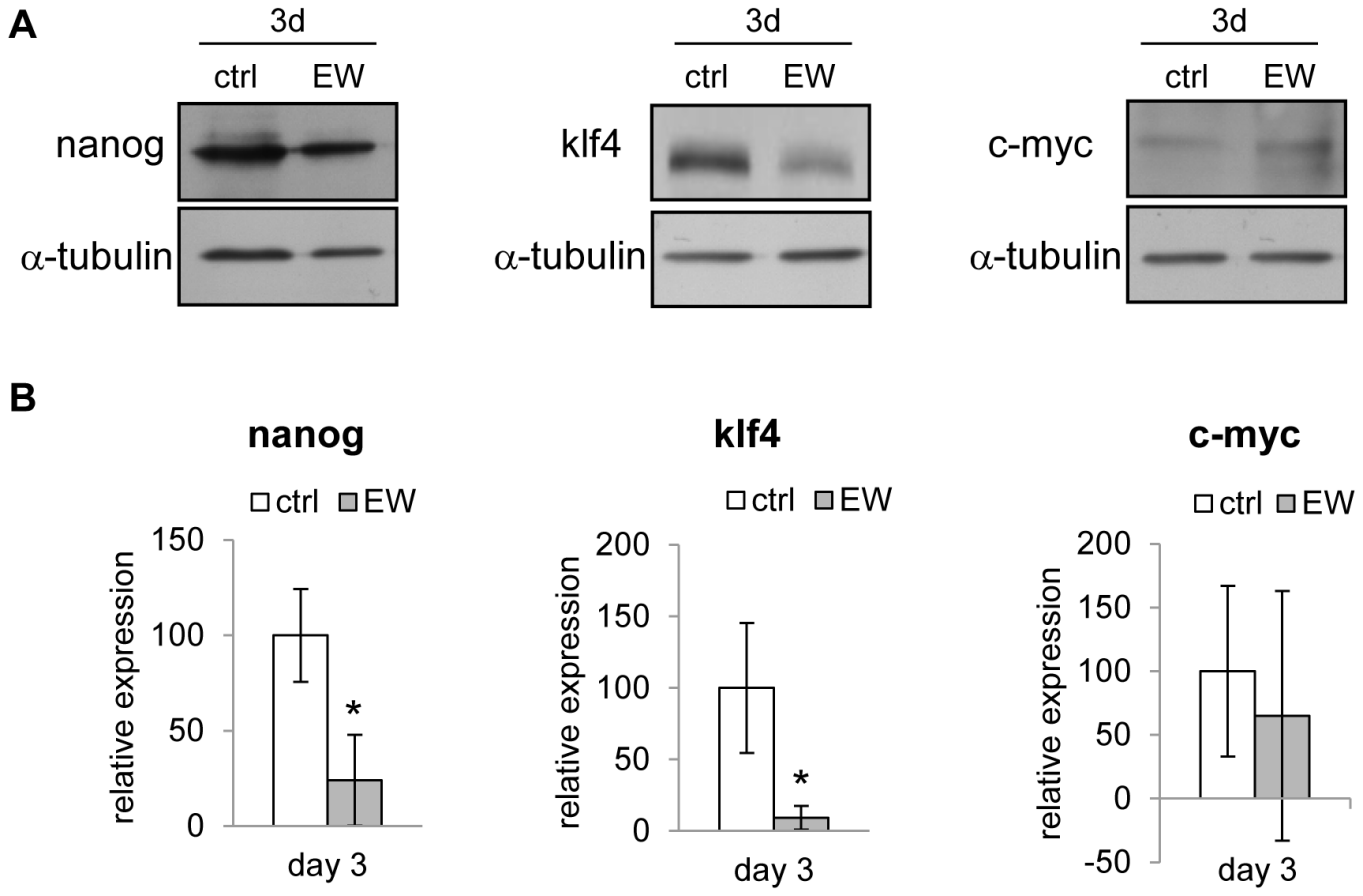
Pluripotency factors expression is influenced by EW. (A) Representative western blot showing the levels of the proteins Nanog, Klf4 and c-Myc in MDA-MB-231 cells, after 3 days of treatment with EW. Alpha tubulin was used as a loading control. (B) Densitometric quantification of Nanog, Klf4 and c-Myc respectively, obtained normalizing the O.D. of protein bands versus the O.D. of α-tubulin bands, chosen as loading control. The results are the mean (SD) of four independent experiments; *p<0.05 vs ctrl by Student’s *t* test.

In MDA-MB-231 specimens oct4 and sox2 proteins have been not detected, whereas lower levels of Klf4, c-Myc and Nanog have been recorded in EW-treated samples after three days. That down-regulation attains statistically significance only for Klf4 and Nanog factors (Fig. 7).

### Ovalbumin Effects

OV did not cause significant difference in cell number of both MCF-7 and MDA-MB-231 cells, in respect to control samples (not shown).

**Figure 8:**
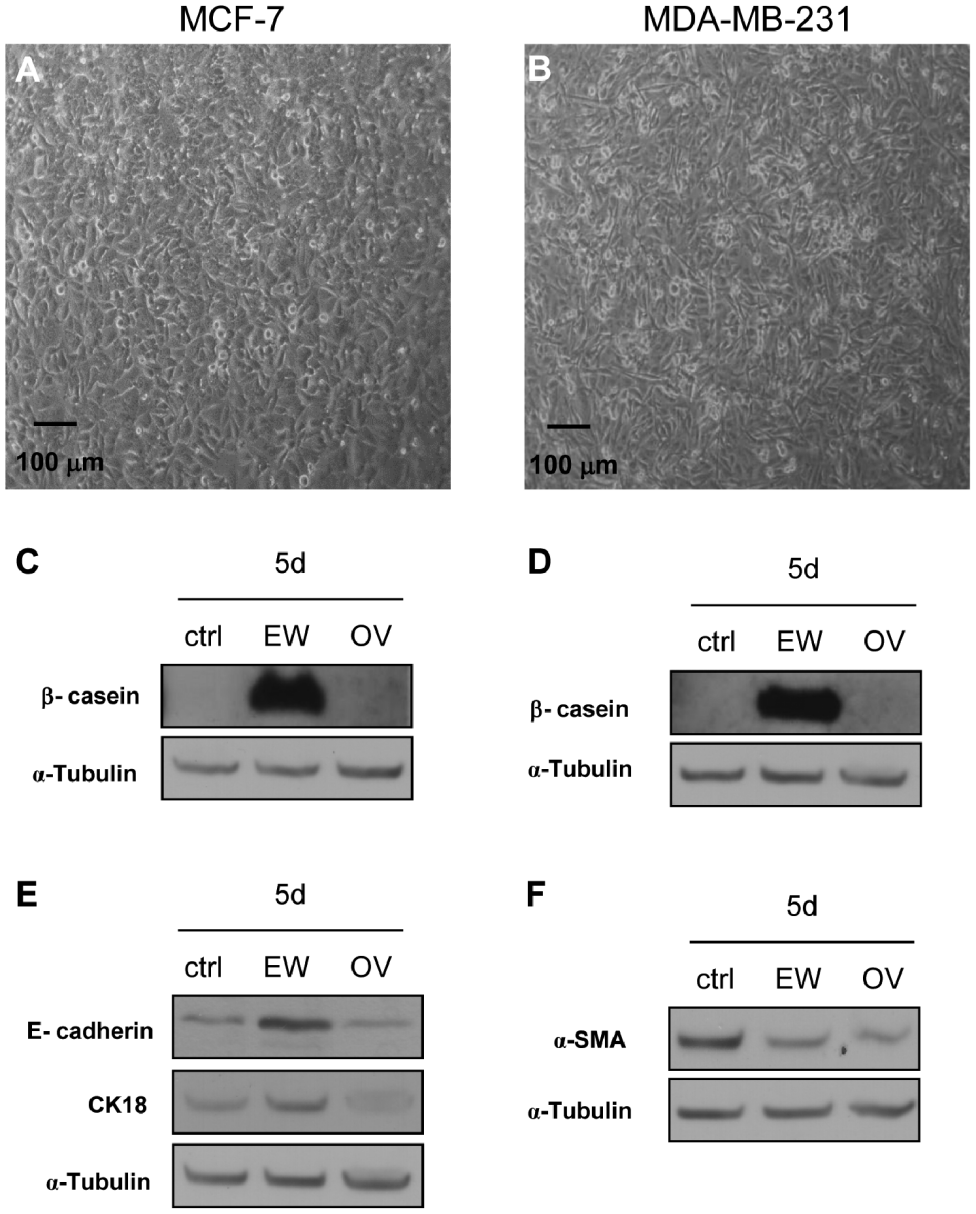
Ovalbumin is able to downregulate α-SMA biosynthesis in MDA-MB-231 cells. Phase contrast microscopy. (A): confluent MCF-7 cells in OV condition (day 7). (B): confluent MDA-MB-231 cells in OV condition (day 7): OV-treated cells did not form acini or duct-like structures in both cell lines. (C) and (D): representative western blot showing the levels of the protein β-casein in MCF-7 and MDA-MB-231 cells respectively, after 5 days. (E) and (F): representative western blot showing the levels of the proteins E-cadherin, CK18, and α-SMA in MDA-MB-231 cells, after 5 days; α-tubulin was used as a loading control. Ctrl (control), EW (egg white), and OV (ovalbumin).

We also found that OV did not determine the formation of three-dimensional structures in both MCF-7 than in MDA-MB-231 after 7 days of treatment (Fig. 8A and 8B). Moreover, unlike the EW, OV did not stimulate the biosynthesis of β-casein neither in MCF-7 nor in MDA-MB-231 cells (Fig. 8C and 8D). With regard to the epithelial markers, OV had no effect on protein biosynthesis of E-cadherin and CK18 (Fig. 8E); on the contrary, similarly to EW, OV decreased the mesenchymal marker α-SMA (Fig. 8F).

## Discussion

Several studies have brought to light the relevance of embryonic microenvironment in suppressing the tumorigenic phenotype of malignant cancer cells through a complex and still unidentified set of biological pathways, involving gene regulation, induction of apoptosis, inhibition of proliferation and modulation of morphogens release [19]; [37]–[39].

Herein we investigated how a microenvironment of maternal origin could lead to the reversion of the cancer phenotype.

Data collected on two different breast cancer cell lines support the hypothesis that EW triggers the *in vitro* reversion of malignant phenotype, confirming previous observations about the capability of embryonic/maternal microenvironment to revert cancer cells [25]. This is a quite astonishing result, as morphological reversion of malignant phenotype has been hitherto successfully achieved only on 3D-culture [40].

MCF-7 cells cultured in EW developed acini and mammary duct-like structures, after a transient growth effect. This result should not be surprising, given that duct initiation requires a preliminary proliferation phase [41]. Indeed, that effect was not observed in MDA-MB-231 cells, which are recruited to form only simple buds of mammary structures.

Commitment of breast cancer cells into a differentiating process requires to be supported by the formation of complex mammary structures, followed by their cavitation. Kenny and colleagues classified 25 breast cancer cell lines depending on their ability to form specific structures in 3D cultures [42]: only epithelial mammary normal cells are able to form hollow round structures in Matrigel™, whereas MCF-7 and MDA-MB-231 cells are assigned respectively to the mass and the stellate categories, because they form respectively full and disorganized structures, without cavitation.

Surprisingly, MCF-7 cells, even in 2D culture, when conditioned with EW, developed three-dimensional structures, i.e. hollow acini and duct-like structures. Such structures underwent cavitation akin to that described for human immortalized mammary epithelial MCF-10A cells [43]. This “structural” project was only partially sketched in MDA-MB-231 cells growing in EW, thus highlighting the different responsiveness of the two cell lines in presence of the same microenvironmental cues, as previously reported [42].

β-casein is a specific molecular marker of epithelial breast cell functional differentiation [44]. It is noteworthy that a *de novo* synthesis, and a concomitant release of high levels of β-casein, occurred in both MCF-7 and MDA-MB-231 cells cultured in EW. Namely, in MCF-7 cells, confocal microscopy shows that β-casein staining is localized into the lumen of the duct, demonstrating that secretion is correctly oriented, as confirmed by TEM images showing polarized MCF-7 cell with secretory vesicles placed in the apical part of the cell, and directed toward the lumen.

Additionally, EW microenvironment was able to counteract the EMT undergone by MDA-MB-231 cells. EMT is a process characterized by loss of cell adhesion, repression of E-cadherin expression, β-catenin delocalization, and increased cell motility, and it is thought as a key-step in metastatic spread [35]. In MDA-MB-231 cells exposed to EW, the biosynthesis of E-cadherin was re-activated, and the localization of β-catenin was shifted from the cytoplasm and the nucleus (where it acts as transcriptional regulator in cell proliferation), to the inner membrane, where β-catenin acts as a cytoskeletal element in epithelial differentiation. Those effects demonstrate the ability of EW in hindering the EMT process in MDA-MB-231 cells, and in fostering cell reversion towards an epithelial-like phenotype. Indeed, the cellular redistribution of β-catenin, and the increased biosynthesis of E-cadherin are crucial factors for the *de novo* remodelling of adherens junctions, and they play an important role in ensuring cell-to-cell adhesiveness. Moreover, CK18 (epithelial marker) and α-SMA (mesenchymal marker) proteins were respectively increased and reduced after EW exposition, hence highlighting how relevant that transition was.

Control MDA-MB-231 cells express low levels of E-cadherin, whereas, in EW-treated cells, E-cadherin was found to be up-regulated. In MDA-MB-231 cells, gene coding for E-cadherin (*cdh1*) is subject to a dynamic and reversible methylation process [34], thus leading to an almost complete suppression of E-cadherin synthesis. On the contrary, EW-exposed cells show a significant biosynthesis of E-cadherin, thus prompting us to hypothesize that EW could epigenetically influence the *cdh1* methylation status. Therefore, we studied the expression and the promoter methylation status of *cdh1* gene at different times. As expected, a significant increase in *cdh1* gene transcription was recorded starting from the fifth day in EW-treated MDA-MB-231 cells. However, the transcriptional increase occurred despite the *cdh1* promoter remained always hyper-methylated, even in EW microenvironment. That finding suggests that mechanisms other than CpG island methylation may be involved in the up-regulation of *cdh1* gene. The microRNA-200 family is involved in *cdh1* regulation, through direct targeting of transcriptional repressors, such as ZEB1 and ZEB2 [35]. However, we did not find any differences in ZEB1 and ZEB2 levels, in both control and EW-treated MDA-MB-231 cells. Thus, we focused on alternative mechanisms of *cdh1* gene regulation. Several lines of evidence suggest that histone methylation, especially histone H3 trimethylation at lysine 4, may be positively associated with the transcriptional activation of the *cdh1* gene [45]. Hence, we investigated the methylation status of histone H3 in MDA-MB-231 cells. We found the extent of histone H3 trimethylation at Lys 4 in EW-treated MDA-MB-231 cells 1.8 times greater than in MDA-MB-231 control cells after 5 days, just as *cdh1* begins to be more expressed. A pivotal role in histone H3 methylation is sustained by a multi-protein complex, in which LSD1 and SNAIL proteins are crucial factors. LSD1 is a lysine-specific demethylase 1 interacting with SNAIL, a specific zinc-finger transcription repressor of the *cdh1* gene [45]. When SNAIL is expressed in a cell population, the LSD1-SNAIL ratio could be thought as an indicator of the effectiveness of the histone demethylating complex. Thus, we investigated LSD1-SNAIL levels, and, as expected, we found that ratio significantly decreased in EW-cultured MDA-MB-231 cells. The decrease of LSD1 may indeed rescue the transcription of genes regulated by Histone 3 lysine 4 methylation status. *Cdh1* belongs to this group of genes: as a result, by increasing histone H3 Lys 4 methylation status, the *cdh1* transcription and E-cadherin biosynthesis should likely be reactivated in EW-exposed MDA-MB-231 cells.

Finally, we evaluated in MDA-MB-231 cells whether the EW-induced differentiation could be considered a reprogramming process. Cell reprogramming is the process leading, normal or cancerous cells, to become iPSCs, i.e. recovering staminality [28]. Transcription factors Oct4, Sox2, Klf4, c-Myc, and Nanog constitute a self-organizing transcription factor network that prevents the iPSCs differentiation, and promotes proliferation and epigenetic processes required for pluripotency. That process requires the canonical reprogramming genes have to be stably overexpressed [46].

Our results show that the differentiating processes induced by EW in MDA-MB-231 cells are not related to iPSCs generation, given that MDA-MB-231 cells did not express Oct-4 and Sox2; on the contrary, Klf4, c-Myc and Nanog were very weakly expressed in MDA-MB-231 cells, thus evidencing that EW-treatment strongly reduces the endogenous biosynthesis of those factors.

It is however still to be determined whether the effects induced by EW have to be ascribed to specific EW single components, or if that property should be considered a ‘systems property’ of the EW culture medium.

OV constitutes about the 54% of the proteins of albumen. We hypothesized that some effects induced by the EW could be determined by OV alone; therefore we studied whether such protein could exert an anti-proliferative effect, akin to that demonstrated for human serum albumin (estrocolyone-I) [47]. Our results rule out this hypothesis because treatment with OV did not cause any significant difference neither in cell number nor in morphological/functional differentiation, when compared to controls. However, OV reduced α-SMA levels, and left unchanged the epithelial markers. From these results it can be inferred that EW activities herein reported could be ascribed to a “systems-property”, given that the individual components of the EW may justify only some of the effects shown by the EW in its entirety.

It is still a matter of study to ascertain if the “reverting” properties of EW are driven by the biophysical features of that microenvironment. We demonstrated that in EW-3D-like microenvironment relevant changes occurred in the way cells interact with each other. The interactions between cells, and between cells and their substratum enact profound modifications in cell shape and function, as documented by several studies [25], [48]–[50]. Preliminary results (not shown) obtained in our laboratory indicate that EW is able to influence the biophysics of cells: indeed, the elastic module, measured through atomic force microscopy, in both MCF7 and MDA-MB-231 cells, was significantly increased from 20% to 40%, after 7 days of culture in EW. As a result, cancer EW-treated cells resulted less deformable and less elastic, thereby indicating that cancer cells have lost some of the biophysical characteristics of malignancy [51]. Overall, such results suggest that EW-mediated cancer reversion should be viewed as a consequence of deep modification in the interplay between cancer cells and their microenvironment, as it has been repeatedly outlined by the Tissue Organization Field Theory. Studies on that field are currently in progress in our laboratory.

## Supporting Information

Video S1 **β-catenin in EW-treated MCF-7 cells.** The video reports a spatial series of optical sections, obtained with confocal microscopy, relative to the formation of hollow duct-like structures in MCF-7 cells, detected with the staining of the membrane perimeter protein β-catenin (green), and of nuclei (blue).
(AVI)

Video S2 **β-casein in EW-treated MCF-7 cells.** The video reports a spatial series of optical sections, obtained with confocal microscopy, showing that the secretion of β-casein (red) is correctly oriented towards the lumen of acini and duct-like structures in MCF-7 cells. Nuclei are stained in blue.
(AVI)
