# Expression of Concern: Microenvironment Promotes Tumor Cell Reprogramming in Human Breast Cancer Cell Lines

**DOI:** 10.1371/journal.pone.0217961

**Published:** 2019-05-31

**Authors:** The PLOS ONE Editors

After publication of this article [1], the following concerns were raised:

- The α-tubulin panels appear similar in Fig 4A, Fig 4B. The original images underlying these panels are no longer available, but the authors provided original images which they noted were obtained using different exposures of the same β-casein and α-tubulin blots, in S1 File. The shapes of the bands in the α-tubulin blots provided do not appear to match those in the published figure panels.
- In Fig 5C the background in the α-tubulin panel is unexpectedly uniform. The authors commented that this is an accurate reflection of the original image data. Images of the same blots obtained using a different exposure are included here in S2 File, both for ZEB1 and α-tubulin.
- In Fig 6C there appear to be haloes around the bands in the CK18 panel. This is also seen on the original blot image provided (S3 File), which represents the same blot imaged at a different exposure than the published image. S3 File also includes replicate data to support the α-SMA and α-tubulin results in Fig 6C; the original images supporting these panels are no longer available.
- In Fig 7A there are similarities between the α-tubulin data shown in lane 1 of the middle and lane 2 of the right panel. The primary image data needed to resolve this concern are no longer available. The authors have provided replicate data confirming the Fig 7A results in S4 File.

The authors clarified that other original data underlying the results reported in this article, including the quantification of the western blot results in the above figures, are no longer available.

In light of the concerns raised regarding multiple figure panels and the unavailability of image data supporting some results in question, the *PLOS ONE* Editors issue this Expression of Concern.

## Supporting information

S1 File Image data supporting Fig 4. The original blots related to the bands shown in the article are lacking. This file includes images of the same blots at different exposure times, confirming the published data. Signals related to MCF7 cells are in the left side of the left blots (1A); signals related to MDA cells are in the left side of the right blots (1B); β-casein in the upper blots, α-tubulin in the lower.
(PDF)

S2 File Image data supporting Fig 5C. The original blots related to the bands shown in the article are lacking. This file includes images of the same blots at different exposure times. ZEB1 is showed in the upper blot, left side; α-tubulin in the lower blot. “C3-W3” and “C5-W5” are referred to control and EW, day 3 and day 5, respectively.
(PDF)

S3 File Image data supporting Fig 6C. For CK18, a different exposure of the same blot reported in the article is provided (left blot); the halo is visible around all the signals. For α-SMA (center blot) and α-tubulin (right blot), replicate western blot images are provided. The bands of interest are indicated by an arrow. The lanes C5 and W5 are referred to control and EW, day 5, respectively.
(PDF)

S4 File Image data supporting Fig 7A. Replicate data from parallel experiments are provided. Samples are arranged as “4 ctrl-4 EW”, i.e. the first four left lanes include control samples and the 4 right lanes include EW samples. The specific bands are evidenced by arrows: nanog, upper, left; klf4, upper, right; c-myc, lower, left; α-tubulin, lower, right.
(PDF)
