# Supplementary figures and images for: Expression of Concern: Microenvironment Promotes Tumor Cell Reprogramming in Human Breast Cancer Cell Lines
Source: PLoS One. 2019 May 31;14(5):e0217961. doi: 10.1371/journal.pone.0217961 (PMC6544323; doi:10.1371/journal.pone.0217961)

# QUERY 1 - FIG 4

MCF 7

$\beta$ -CASEIN

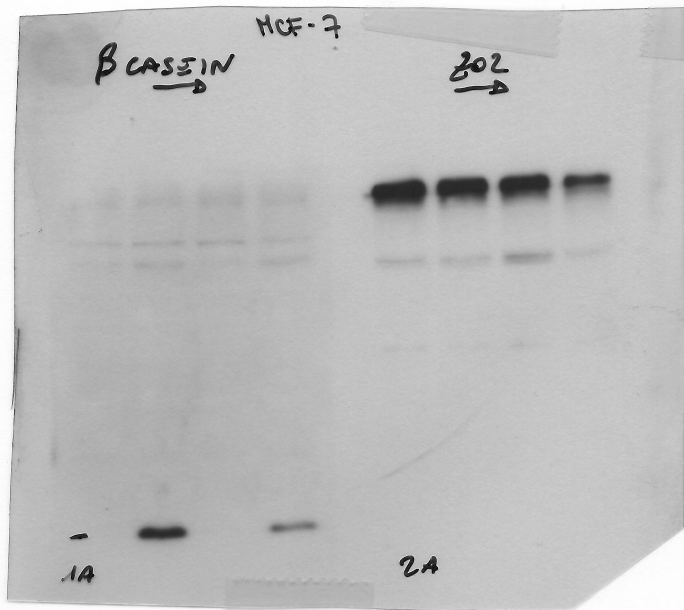

MDA

$\beta$  CASEIN

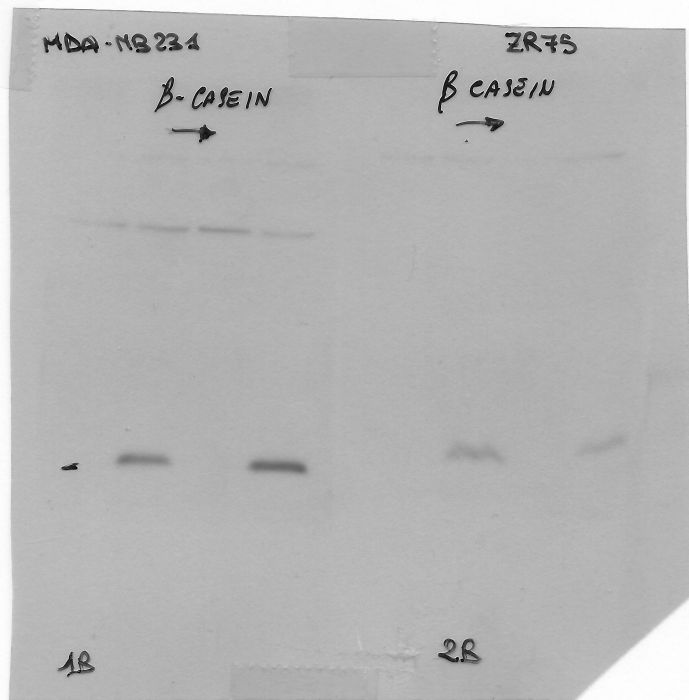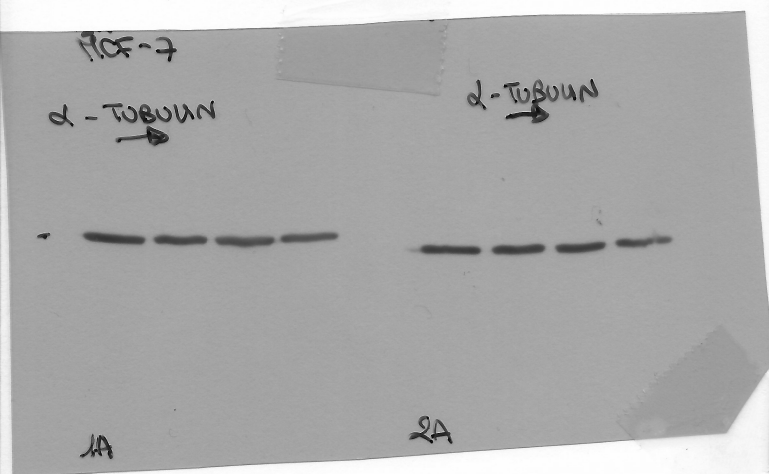

$\alpha$ -TUBULIN

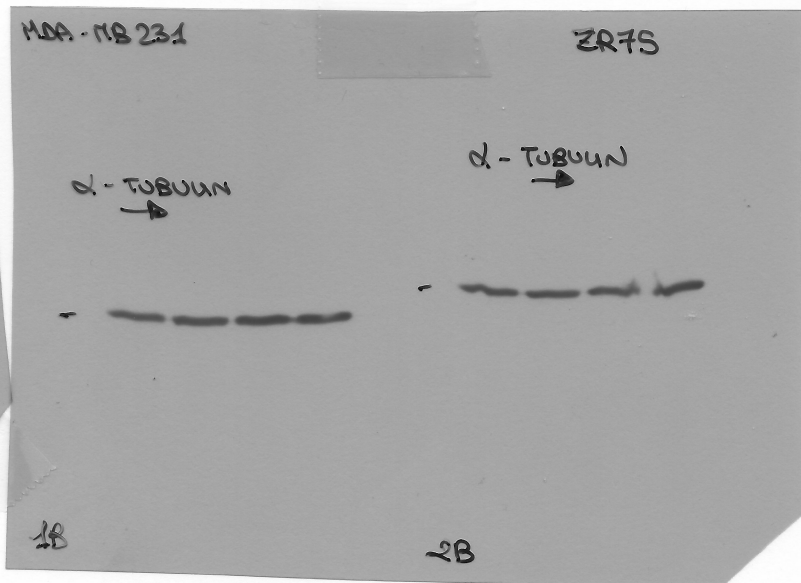

$\alpha$ -TUBULIN

Supplement: S1 File — The original blots related to the bands shown in the article are lacking. This file includes images of the same blots at different exposure times, confirming the published data. Signals related to MCF7 cells are in the left side of the left blots (1A); signals related to MDA cells are in the left side of the right blots (1B); β-casein in the upper blots, α-tubulin in the lower. (PDF) [file pone.0217961.s001.pdf]

QUERY 2 - FIG 5

ZEB1

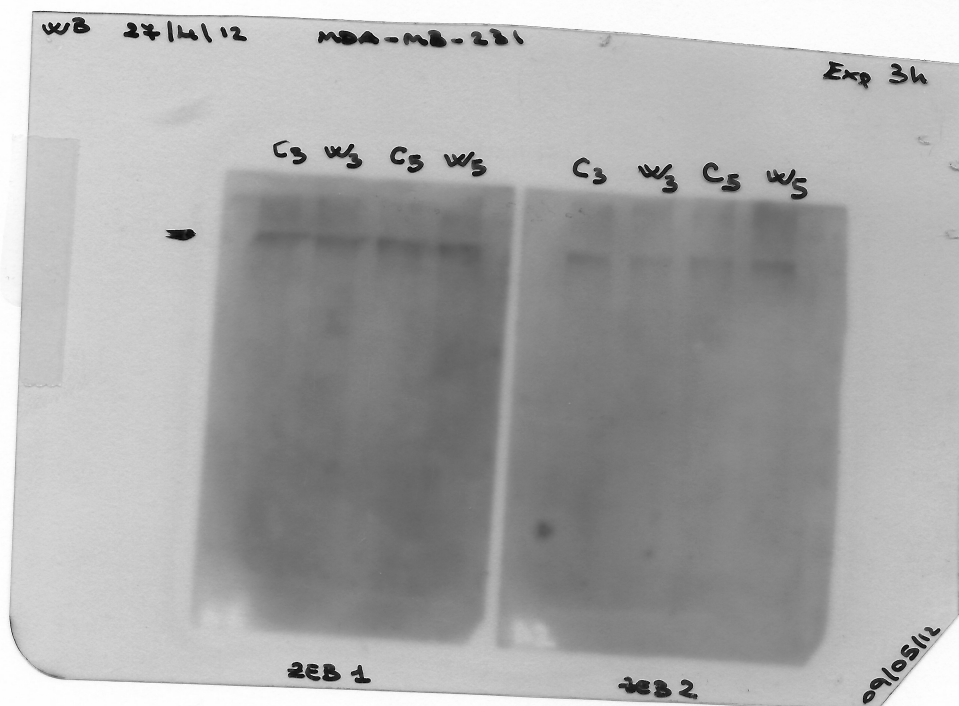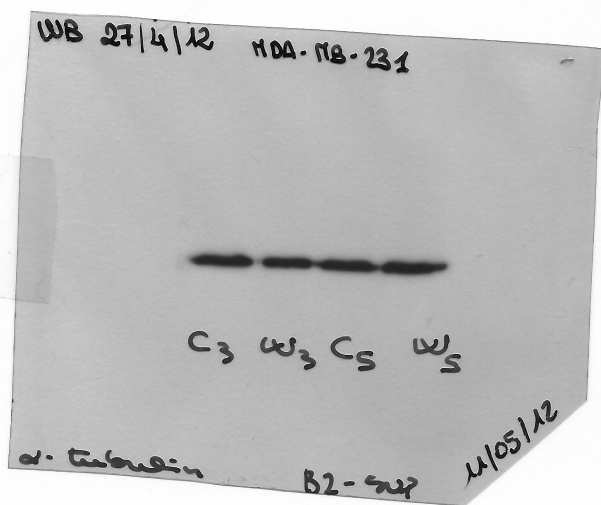

$\alpha$ -TUBULIN

Supplement: S2 File — The original blots related to the bands shown in the article are lacking. This file includes images of the same blots at different exposure times. ZEB1 is showed in the upper blot, left side; α-tubulin in the lower blot. “C3-W3” and “C5-W5” are referred to control and EW, day 3 and day 5, respectively. (PDF) [file pone.0217961.s002.pdf]

QUERY 4 - FIG 7 A

## DIFFERENT EXPERIMENT (WB) 4 CONTROLS - 4 TREATED

NANOOG

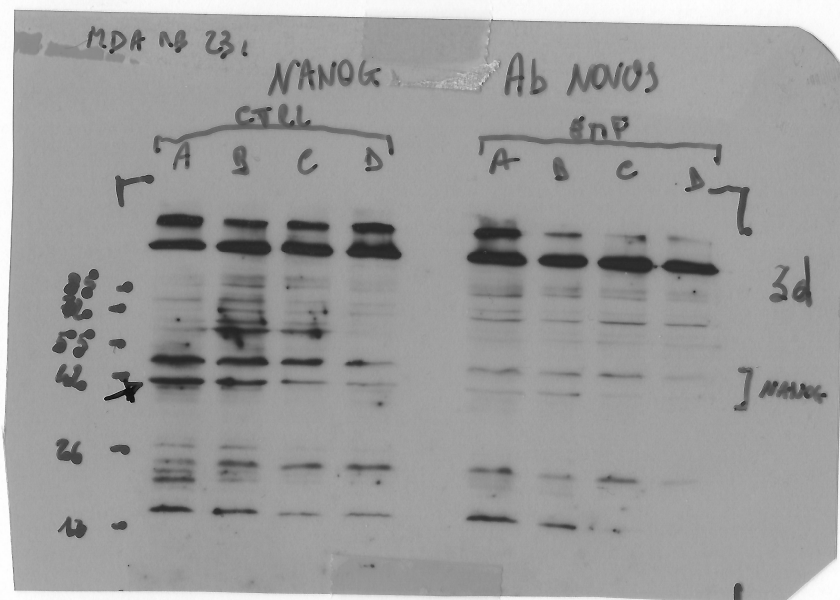

KLPH

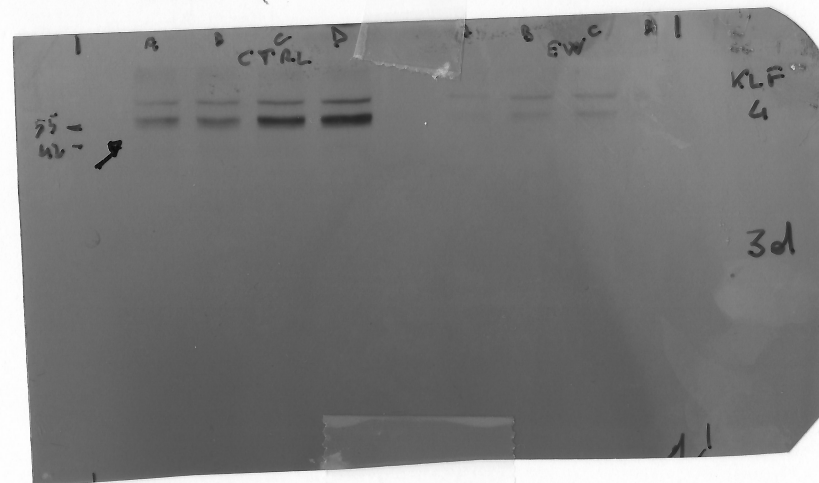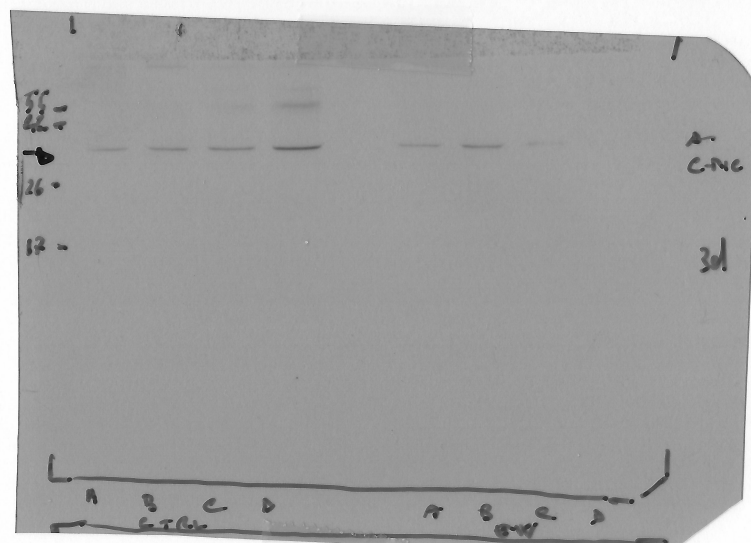

C-MYC

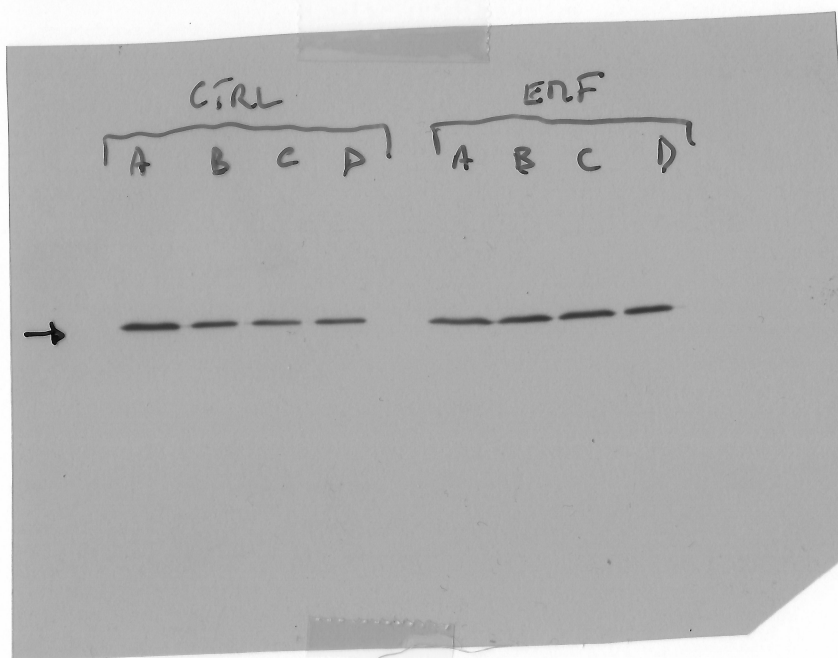

2-TUBULIN

Supplement: S4 File — Replicate data from parallel experiments are provided. Samples are arranged as “4 ctrl-4 EW”, i.e. the first four left lanes include control samples and the 4 right lanes include EW samples. The specific bands are evidenced by arrows: nanog, upper, left; klf4, upper, right; c-myc, lower, left; α-tubulin, lower, right. (PDF) [file pone.0217961.s004.pdf]
