# Supplementary material for: Expression of Concern: Microenvironment Promotes Tumor Cell Reprogramming in Human Breast Cancer Cell Lines
Source: PLoS One. 2019 May 31;14(5):e0217961. doi: 10.1371/journal.pone.0217961 (PMC6544323; doi:10.1371/journal.pone.0217961)

QUERY 3 - FIG 6C

CK18
SAME MEMBRANE
DIFFERENT EXPOSITION

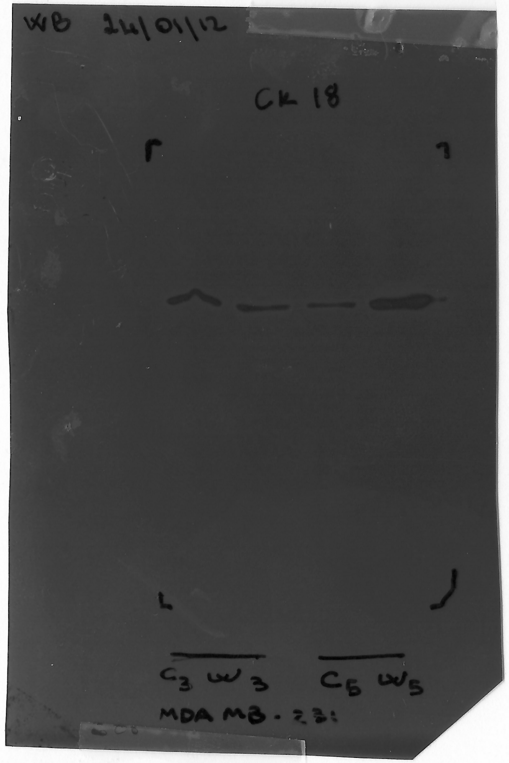

α - SMA
DIFFERENT EXPERIMENT (WB)

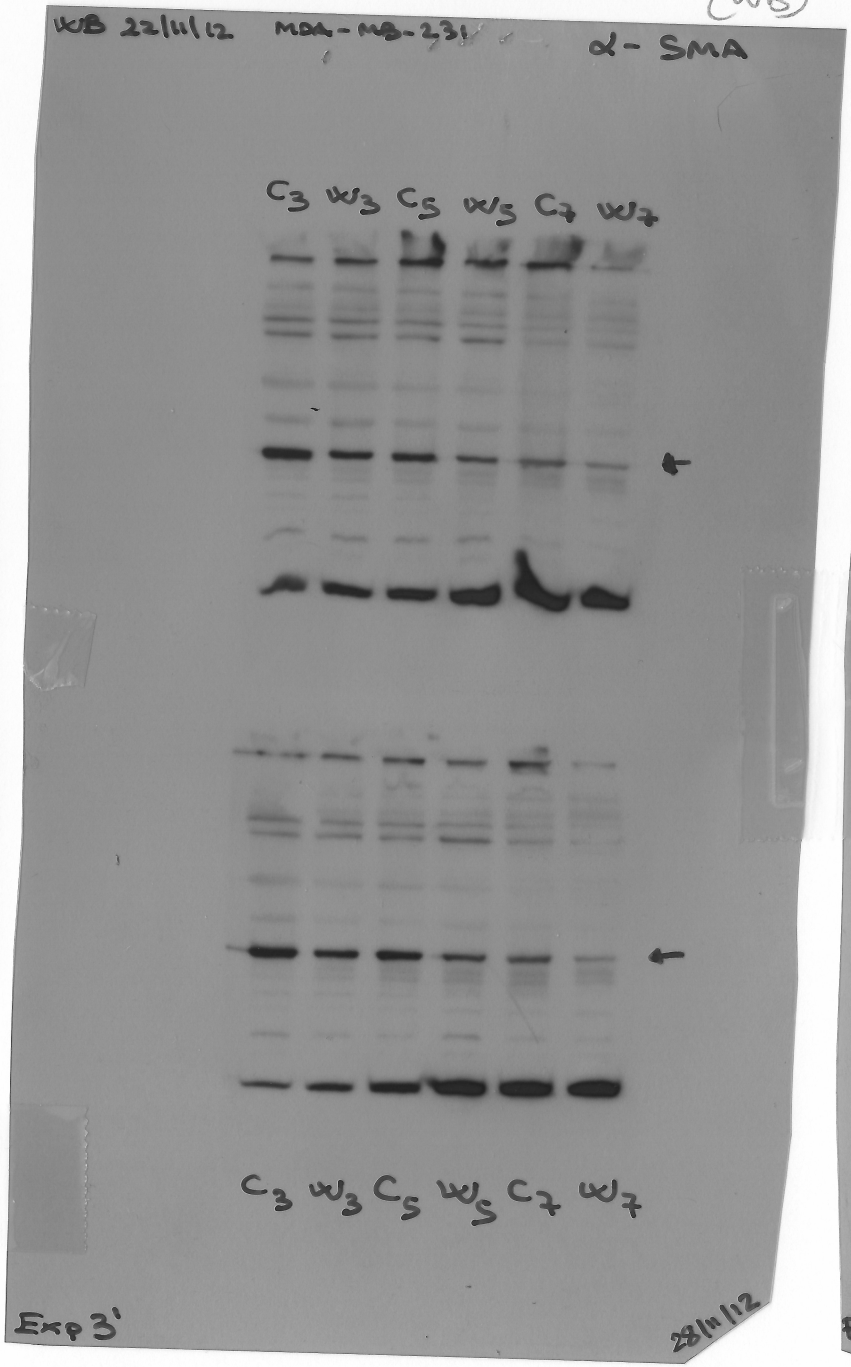

α-TUBULIN
DIFFERENT EXPERIMENT (WB)

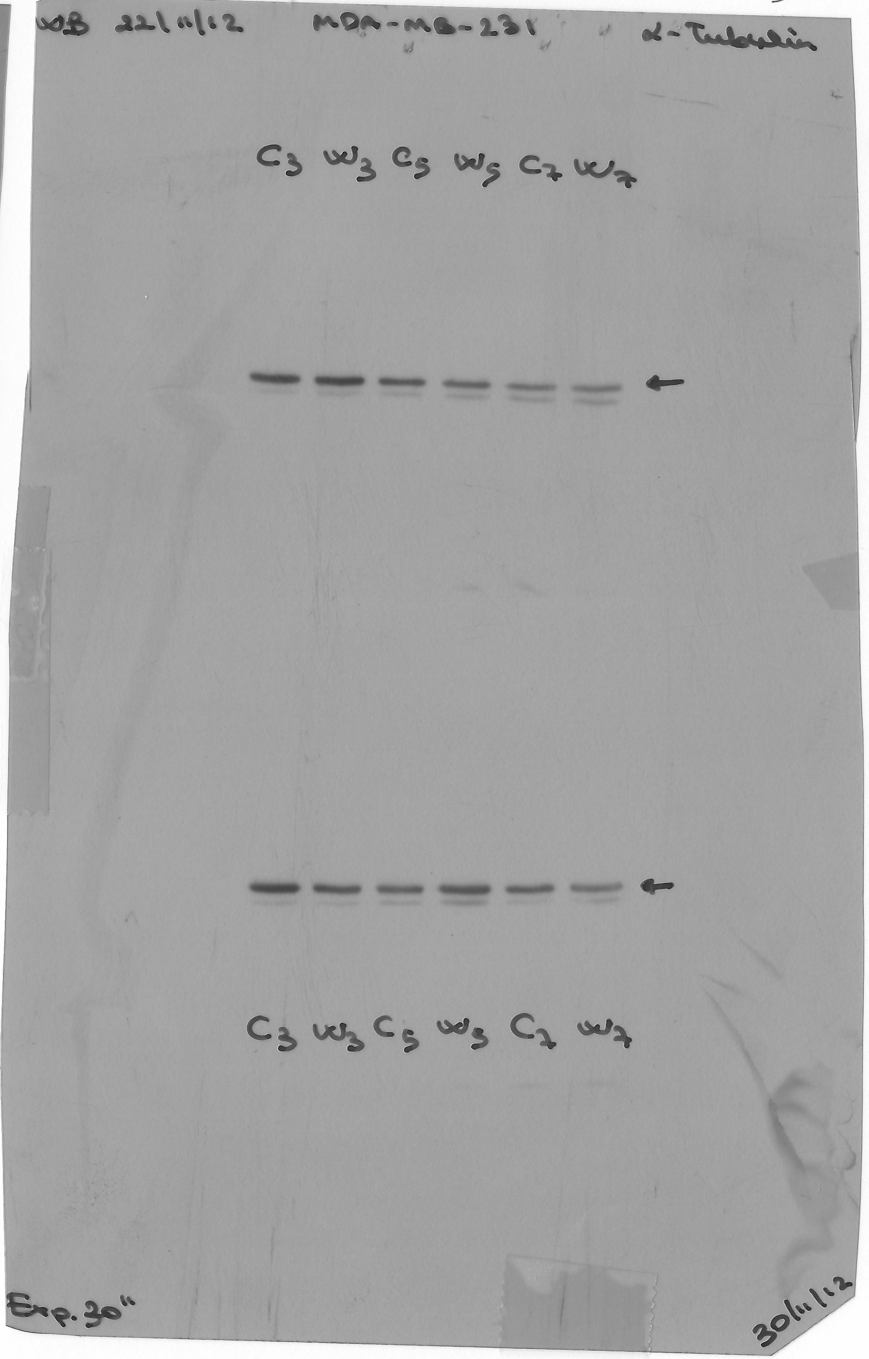

Supplement: S3 File — For CK18, a different exposure of the same blot reported in the article is provided (left blot); the halo is visible around all the signals. For α-SMA (center blot) and α-tubulin (right blot), replicate western blot images are provided. The bands of interest are indicated by an arrow. The lanes C5 and W5 are referred to control and EW, day 5, respectively. (PDF) [file pone.0217961.s003.pdf]
